# Proapoptotic function of deubiquitinase *DUSP31* in *Drosophila*

**DOI:** 10.18632/oncotarget.19715

**Published:** 2017-07-31

**Authors:** Sergey A. Sinenko

**Affiliations:** ^1^ Department of Pharmacology and Cancer Biology, Duke University Medical Center, Durham, NC 27710, USA; ^2^ Institute of Cytology, Russian Academy of Sciences, Saint Petersburg 194064, Russia

**Keywords:** apoptosis, deubitiquinase, DUSP31, Dark, Drosophila

## Abstract

*Drosophila* have been used to identify new components in apoptosis regulation. The *Drosophila* protein Dark forms an octameric apoptosome complex that induces the initiator caspase Dronc to trigger the caspase cell death pathway and, therefore, plays an important role in controlling apoptosis. Caspases and Dark are constantly expressed in cells, but their activity is blocked by DIAP1 E3 ligase-mediated ubiquitination and subsequent inactivation or proteasomal degradation. One of the regulatory mechanisms that stabilize proapoptotic factors is the removal of ubiquitin chains by deubiquitinases. In this study performed a modified genetic screen for deubiquitinases (dsRNA lines) to identify those involved in stabilizing proapoptotic components. Loss-of-function alleles of deubiquitinase *DUSP31* were identified as suppressors of the Dronc overexpression phenotype. *DUSP31* deficiency also suppresses apoptosis induced by the RHG protein, Grim. Genetic analysis revealed for the first time that *DUSP31* deficiency sufficiently suppresses the Dark phenotype, indicating its involvement in the control of Dark/Dronc apoptosome function in invertebrate apoptosis.

## INTRODUCTION

Apoptosis or programmed cell death is an important process in maintaining proper development and tissue homeostasis in multicellular organisms [1]. The apoptotic program is triggered by signaling events that result in activation of cysteine proteases called caspases. Eukaryotic cells constitutively express all components of apoptotic machinery, and caspase activation must be regulated by a very precise and sensitive mechanism. Proteolytic cascades mediated via activator dimerization cleave initiator caspases to activate effector caspases, which consequentially cleave multiple protein substrates, ultimately advancing the cell to destruction [2, 3]. In vertebrates, the core programmed cell death pathway is mediated via cytochrome-c release from the mitochondria which activates adaptor protein Apaf-1 to form a heptameric apoptosome complex [4, 5]. The Apaf-1 apoptosome complex facilitates autocatalytic cleavage of the caspase-9 zymogen and, as such, exhibits two orders of magnitude higher catalytic activity than free caspase-9 [6–8]. However, in *Drosophila*, the analogous pathway appears to have no dependence on free cytochrome-c [9–11]. Instead, the *Drosophila* Apaf-1 homolog, death-associated Apaf1-related killer (Dark) is permanently expressed and can form an octameric apoptosome complex which then activates the key initiator caspase death regulator Nedd2-like caspase (Dronc) [12–14]. Unlike caspase-9, the activated Dronc caspase domain is dissociated from the apoptosome but exhibits robust protease activity toward effector caspases [15–17]. Initiation of apoptosis in *Drosophila* is solely dependent on caspase inhibitors known as inhibitor of apoptosis proteins (IAPs) [18, 19]. A major *Drosophila* IAP, DIAP1, is an E3 ligase whose activity mediates the anti-apoptotic function via ubiquitination of caspases [16, 20, 21]. This protein is constantly expressed in *Drosophila* cells and eliminates active caspases, either inducing their degradation [22] or acting via nondegradative mechanisms [23–26]. Apoptotic stimuli block DIAP1’s activity mainly via transcriptional activation of a family of IAP antagonists, including Reaper (Rpr), Hid, and Grim (RHG proteins or IAP antagonists) [27–29], which bind DIAP1’s BIR domains, preventing their interaction with caspases [30, 31]. Contrary to DIAP1, much less is known about how other central proapoptotic protein, Dark, and the related apoptosome complex are regulated in *Drosophila* [12, 32]. It is hypothesized that Dark can also be regulated by ubiquitination, leading to its subsequent inactivation or degradation [15]. The process of protein elimination is often antagonized by specific deubiquitinases (DUBs) which remove the ubiquitin chains from target proteins, thereby stabilizing their activity.

Based on domain structure prediction, there are about 100 highly conserved human proteins which possess deubiquitinating activity, and they can be classified into six subfamilies [33, 34]. They are structurally diverse isopeptidases that specifically cleave ubiquitin conjugates at the ubiquitin carboxyl end. DUBs mediate the maintenance of the free ubiquitin pool in cells by processing ubiquitin precursors and recycling ubiquitin from proteins committed to proteasomal elimination [33]. An important function of many DUBs is to modulate protein stability by removing ubiquitin from target proteins. DUBs are involved in the regulation of several key apoptotic regulators, including IAPs and Bcl-2 family proteins [35–37]. Recently, it has been shown that deubiquitinase DUBAI stabilizes DIAP1, serving as an additional regulator inhibiting *Drosophila* apoptosis [38]. Deubiquitinase DUBA has also been identified as an enzyme associated with regulation of Dronc function [39]. However, the functions of the majority of predicted DUBs remain unknown. Herein, identification of a novel deubiquitinase, *DUSP31*/*CG30421*, that regulates proapoptotic components of the *Drosophila* cell death pathway is described.

## RESULTS AND DISCUSSION

### Genetic screening identified *CG30421/DUSP31* as a Dronc phenotype suppressor

**Figure 1:**
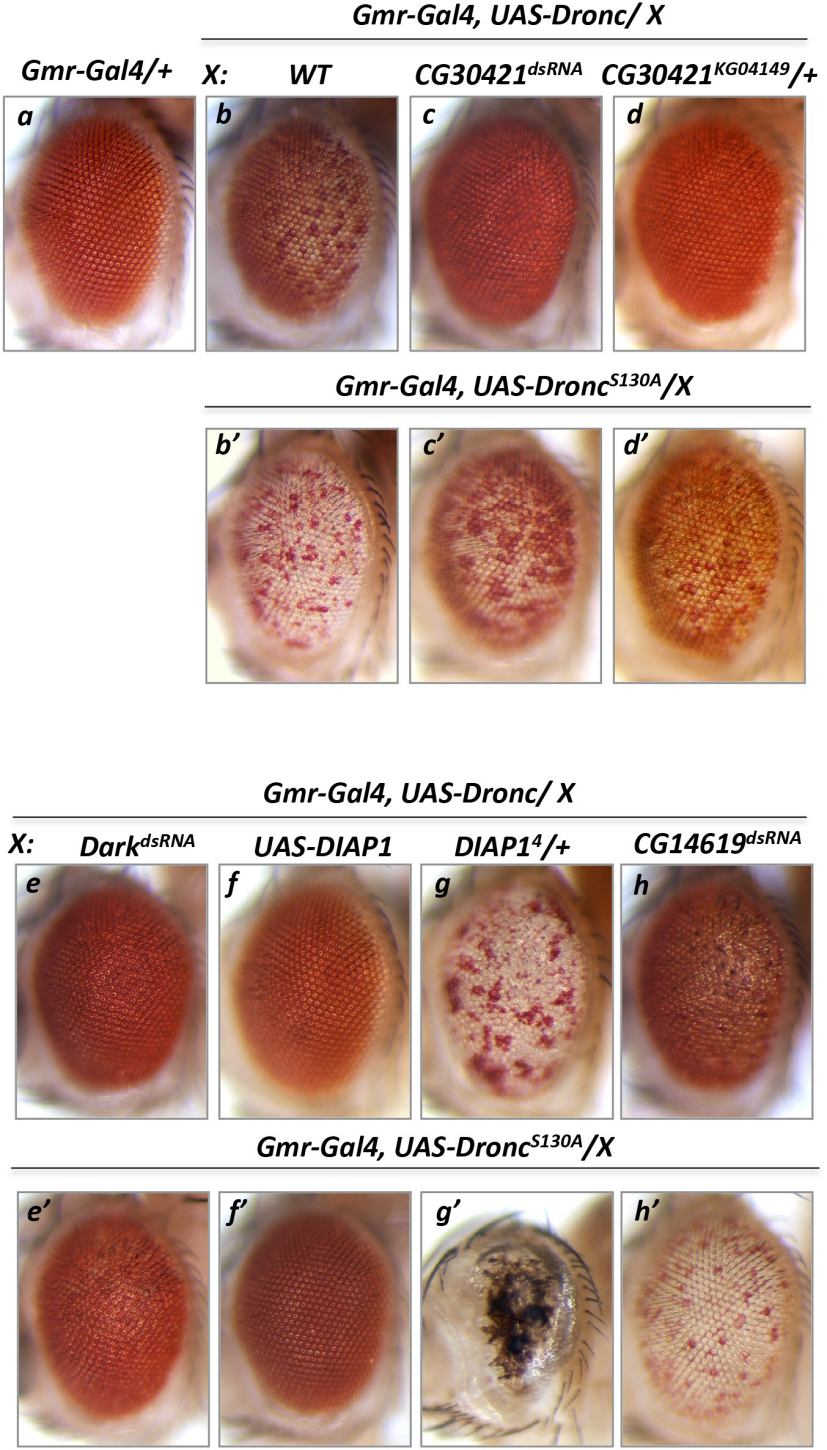
Modifier genetic screen identified deubiquitinase *CG30421* loss-of-function alleles as suppressors of *Dronc* gain-of-function phenotype Overexpression of Dronc induced apoptosis seen as depigmentation of a large number of *Drosophila* eye facets (**b**, *Gmr-Gal4/+*, *UAS-Dronc/+*) compared to *Gmr-Gal4/+* control eyes **(a)**. dsRNA-mediated inactivation of *DUSP31* (**c**, *Gmr-Gal4/UAS-DUSP31*^*dsRNA*^*, UAS-Dronc/+*) or the heterozygous *DUSP31* allele (**d**, *Gmr-Gal4/DUSP31*^*KG04149*^, *UAS-Dronc/+*), but not dsRNA-mediated inactivation of *CG14619* (**h**, *Gmr-Gal4/UAS-CG14619*^*dsRNA*^, *UAS-Dronc/+*), suppressed the apoptotic *Dronc* phenotype in eye photoreceptor cells (b, *Gmr-Gal4/+*, *UAS-Dronc/+*). Phospho-mutant *Dronc*^*S130A*^ induced stronger cell death seen as increased eye depigmentation (**b’**, *Gmr-Gal4/+*, *UAS-Dronc*^*S130A*^*/+*). Similar to wild type *Dronc*, inactivation of *DUSP31* (**c’**, *Gmr-Gal4/ UAS-DUSP31*^*dsRNA*^, *UAS-Dronc*^*S130A*^*/+*) and the heterozygous allele of *DUSP31* (**d’**, *Gmr-Gal4/DUSP31*^*KG04149*^, *UAS-Dronc*^*S130A*^*/+*), but not inactivation of *CG14619* (**h**’, *Gmr-Gal4/UAS-CG14619*^*dsRNA*^, *UAS-Dronc*^*S130A*^*/+*), substantially suppressed the phenotype induced by the activated *Dronc*^*S130A*^ mutant. In the positive control, dsRNA-mediated inactivation of *Dark* (**e**, *Gmr-Gal4/UAS-Dark*^*dsRNA*^, *UAS-Dronc/+* and **e’**, *Gmr-Gal4/UAS-Dark*^*dsRNA*^, *UAS-Dronc*^*S130A*^*/+*) or overexpression of *DIAP1* (**f**, *Gmr-Gal4/UAS-DIAP1*, *UAS-Dronc/+* and **f’**, *Gmr-Gal4/UAS-DIAP1*, *UAS-Dronc*^*S130A*^*/+*) suppressed *Dronc* and *Dronc*^*S130A*^ overexpression phenotypes, while the heterozygous *DIAP1*^*4*^ allele enhanced *Dronc* (**g**, *Gmr-Gal4/+*, *UAS-Dronc/DIAP1*^*4*^) and *Dronc*^*S130A*^ (**g’**, *Gmr-Gal4/+*, *UAS-Dronc*^*S130A*^*/DIAP1*^*4*^) phenotypes.

**Figure 2:**
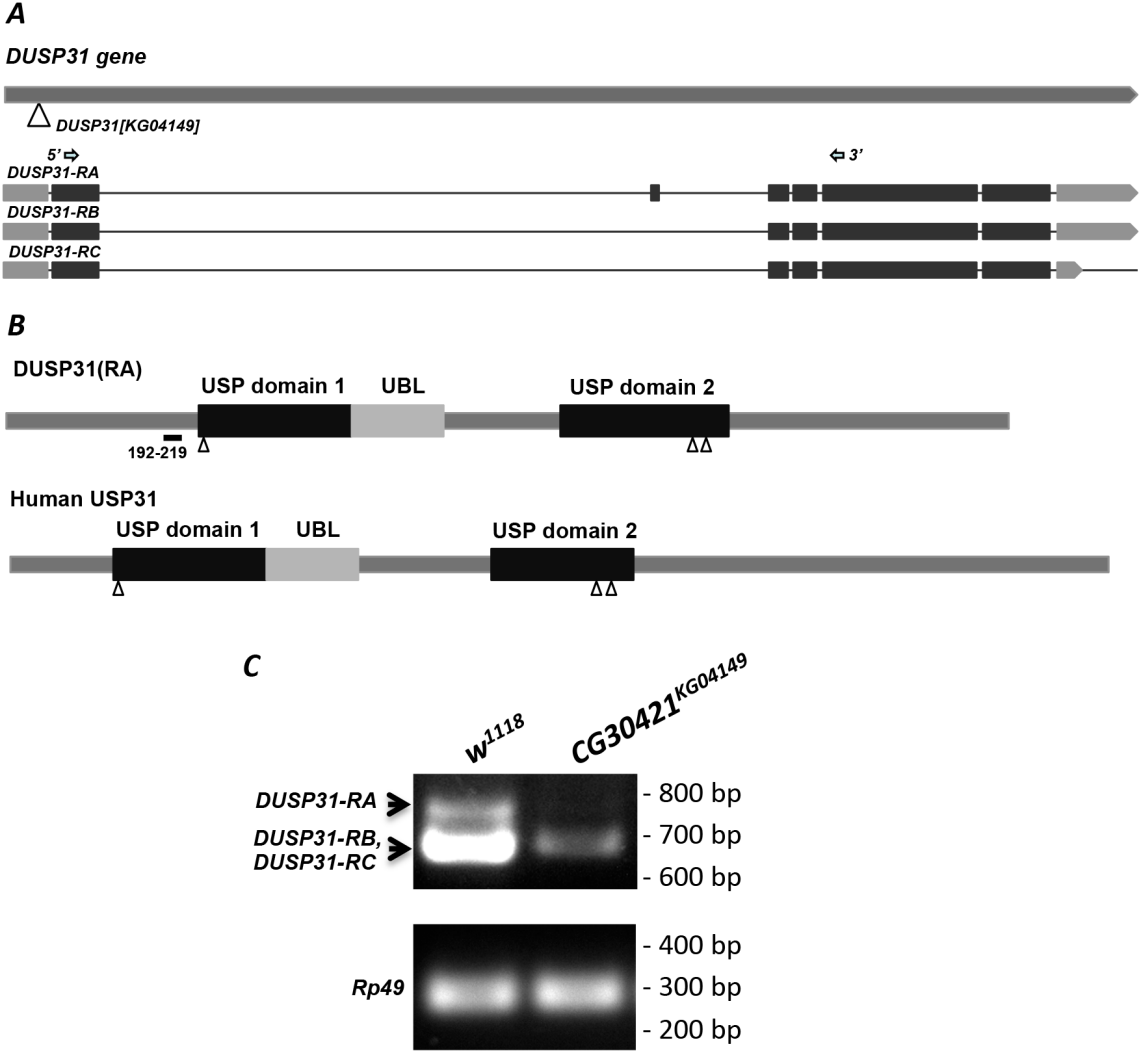
DUSP31^KG04149^ is a hypomorph P-element allele **(A)** Scheme of the *DUSP31* gene. The triangle indicates the location of P-element insertion into the *DUSP31*^*KG04149*^ allele. Three types of mRNA encoding DUSP31-RA, -RB, and -RC are shown (darker boxes are protein coding exons). Arrows indicate locations of forward and reverse primers used for RT-PCR. **(B)** Representation of DUSP31 and human USP31 protein domain structure. These DUBs have two highly conserved USP-catalytic and one ubiquitin-like (UBL) domains. Triangles indicate predicted active sites. The amino acid sequence from 192-219 denotes polypeptide differences between DUSP31-RA and DUSP31-RB/-RC isoforms. **(C)** Abnormal size and significantly reduced mRNA levels of *DUSP31* revealed by semiquantitative RT-PCR of *CG30421*^*KG04149*^ homozygotes compared with wild type tissues (*w*^*1118*^). Arrows indicate amplified fragments of *DUSP31-RA* and *DUSP31-RB/-RC* isoforms. *Rp49* was used as a loading control.

To assess whether any DUB enzymes are involved in regulation of proapoptotic components, such as Dark and Dronc, available double-stranded RNA (dsRNA) lines were screened for their ability to suppress the Dronc gain-of-function phenotype in fly eyes (*GMR-Gal4* driver). Unlike Dark, overexpression of wild type Dronc protein in photoreceptor cells caused a mild apoptotic phenotype featured by an extensive number of depigmented photoreceptor cells (Figure 1b) [32, 40, 41]. Under this condition, Dark tends to be activated by an excess of Dronc, probably via a positive feedback loop triggered by effector caspases [42]. In a control experiment, inactivation of *Dark* in *GMR-Gal4/UAS-Dronc*, *UAS-Dark*^*dsRNA*^*/+* genetic background leads to complete suppression of the *Dronc* phenotype (Figure 1e). Removal of the corresponding Dark or Dronc activity by some unknown factors would be manifested by a reduced number of depigmented eye facets. The modified genetic screen of the available DUBs was designed to identify *UAS-dsRNA* lines able to suppress the *Dronc* phenotype (screen cross: *GMR-Gal4/UAS-Dronc* x *UAS-DUB’s*^*dsRNA*^*/+*). The results of this screen showed the *CG30421* dsRNA line suppresses the *Dronc* phenotype (Figure 1c versus 1b). In addition, the heterozygous *KG04149* allele of this gene could also substantially suppress the *Dronc* phenotype (Figure 1d). Analysis of the *KG04149* allele confirmed that the P-element insertion in the 5’-untranslated region of *CG30421* leads to complete lack of its long isoform expression and significantly reduces that of the short transcripts (Figure 2C and discussed below). None of the other screened DUB dsRNA (16 genes) showed suppression of the *Dronc* phenotype but instead, showed often enhancement, featured by depigmentation and roughness of the eyes (example CG14619, Figure 1h, and Supplementary Table 1). This data suggests that the protein encoded by *CG30421* is able to positively affect some proapoptotic proteins.

Phosphorylation of Dronc at S130 has been shown to mediate suppression of Dronc activation upon metabolic stimulation by glucose-6-phosphate dehydrogenase via increased levels of NADPH [43]. S130 phosphorylation does not affect Dronc’s catalytic activity but inhibits its interaction with Dark [43]. Dronc^S130A^ mutants possess constant and enhanced protease activity (Figure 1b’) [43] that can be substantially suppressed by inactivation of *Dark* (Figure 1e’) and completely suppressed by ectopic *DIAP1* (Figure 1f’) compared with the control genetic background (Figure 1b’). In addition, whether the *CG30421* alleles could also suppress the *Dronc*^*S130A*^ phenotype was also assessed. Importantly, RNA interference-mediated inactivation and the heterozygous *CG30421* allele also substantially suppressed the *Dronc*^*S130A*^ phenotype (Figure 1c’ and 1d’ versus 1b’). Thus, these data additionally suggest that DUBs encoded by *CG30421* are able to positively regulate some proapoptotic proteins. *CG30421* is hereafter referred to as the *Drosophila* ubiquitin specific peptidase 31 (*DUSP31*) gene, as it has the closest homology to mammalian *USP31* (Figure 2B).

Also, inactivation of *DUSP31* or *Dark* was consistently unable to completely suppress the *Dronc*^*S130A*^ phenotype (Figure 1c’ and 1e’), suggesting that some portion of the Dronc protein pool was not dependent on *Dark*’s ability to induce effector caspase activation. Furthermore, the same level of heterozygous DIAP1 (*DIAP1*^*4*^*/+*) potentiated the strongest phospho-mutant *Dronc*^*S130A*^ activity (almost complete loss of eye) compared with mild enhancement of the wild type *Dronc* phenotype by this *DIAP1*^*4*^ allele (Figure 1g’ versus 1g). Thus, *DUSP31* is also required for a positive control of metabolically regulated Dark/Dronc activities.

The level of DUSP31 transcripts is dramatically reduced in *DUSP31*^*KG04149*^ transposon allele homozygotes, as evidenced by RT-PCR analysis (Figure 2C). Accordingly, three predicted transcripts (RA, and almost identical RB and RC) encoding two different protein isoforms of DUSP31 could be amplified (Figure 2A and 2C). The long transcript, DUSP31-RA, is not detected at all, while levels of shorter DUSP31-RB or -RC transcripts are more than 20-times lower in homozygous *DUSP31*^*KG04149*^ flies (Figure 2C), indicating this allele is a strong hypomorph. These homozygous flies are fertile and viable, while ubiquitous inactivation of *DUSP31* with dsRNA (*tub-Gal4*) is lethal, suggesting that there is likely maternal contribution of this gene. Expectedly, expression of *DUSP31*^*dsRNA*^ with *Gmr-Gal4* does not affect eye development.

### *DUSP31* deficiency suppresses Grim-induced apoptosis

**Figure 3:**
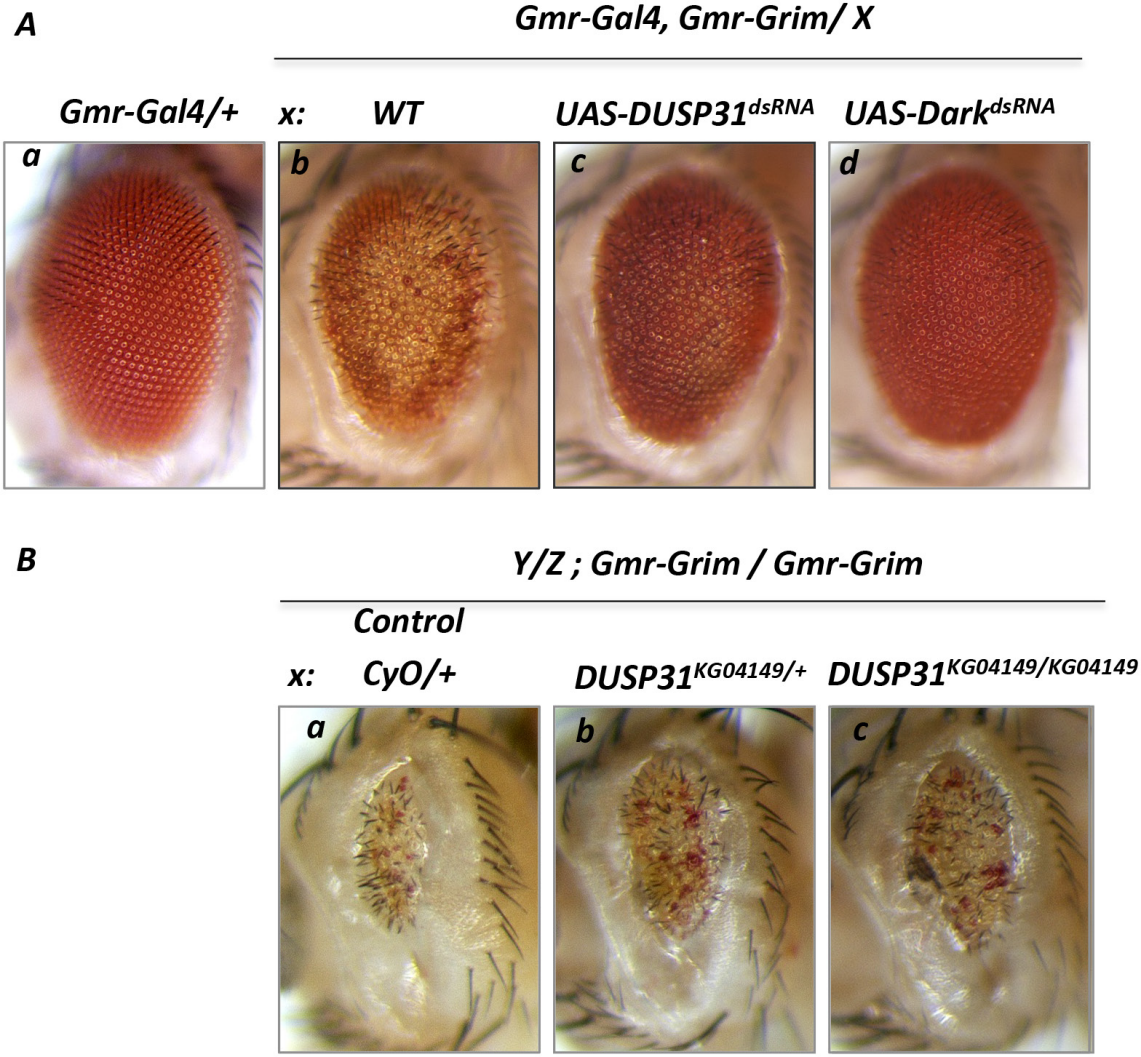
Deficiency in *DUSP31* function suppressed apoptosis induced by overexpression of Grim protein Image of wild type *Drosophila* eye (**Aa**, *Gmr-Gal4/+*). Overexpression of Grim (**Ab**, *Gmr-Gal4/+*, *UAS-Grim/+*) produced a cell death phenotype evidenced by depigmentation and slightly reduced eye size. dsRNA-mediated inactivation of *DUSP31* (**Ac**, *Gmr-Gal4/UAS-DUSP31*^*dsRNA*^, *Gmr-Grim/+*) substantially suppressed depigmentation of eye photoreceptor cells caused by *Grim*-induced apoptosis. Inactivation of *Dark* almost completely suppressed the *Grim*-induced phenotype (**Ad**, *Gmr-Gal4/+*, *Gmr-Grim/UAS-Dark*^*dsRNA*^). **(B)** Deficiency in *DUSP31* only mildly suppressed apoptosis caused by high levels of apoptotic induction. Overexpression of two copies of Grim (Ba, *Gmr-Grim/Gmr-Grim*) produced a strong cell death phenotype characterized by depigmentation, roughness, and a significant reduction in eye size. Heterozygous (Bb, *DUSP31*^*KG04149*^*/CyO*, *Gmr-Grim/Gmr-Grim*) and homozygous (Bc, *DUSP31*^*KG04149*^*/DUSP31*^*KG04149*^, *Gmr-Grim/Gmr-Grim*) *DUSP31* alleles slightly suppressed the *Grim*-induced apoptotic phenotype compared with control (Ba, *CyO/+*, *Gmr-Grim/Gmr-Grim*).

**Figure 4:**
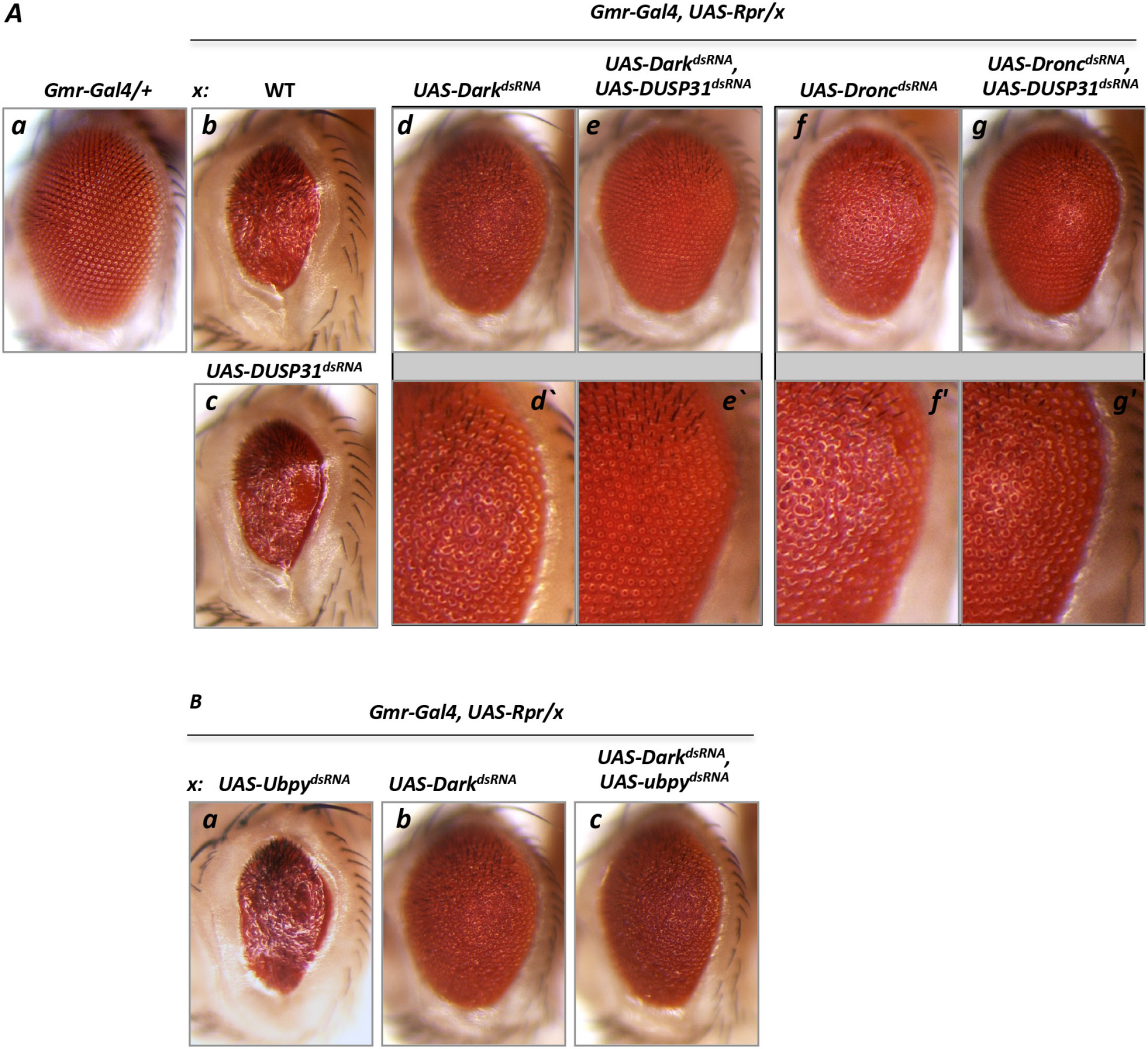
Inactivation of *DUSP31* strongly suppressed the apoptotic phenotype caused by overexpression of *Rpr* and *Dark* dsRNA in *Drosophila* eyes **(A)** Overexpression of *Rpr* (Ab, *Gmr-Gal4/+*, *UAS-Rpr/+*) induced a strong apoptosis phenotype manifested by glassines and a significantly reduced eye size compared with control (Aa, *Gmr-Gal4/+*). Inactivation of *DUSP31* did not suppress the apoptotic phenotype caused by overexpression of *Rpr* (Ac, *Gmr-Gal4/UAS-DUSP31*^*dsRNA*^, *UAS-Rpr/+*). dsRNA-mediated inactivation of *Dark* significantly (but not completely) suppressed the *Rpr*-induced phenotype (Ad, *Gmr-Gal4/UAS-Dark*^*dsRNA*^, *UAS-Rpr/+*) evidenced by glassines and a rough eye phenotype (Ad’). Additional inactivation of *DUSP31* in the *Gmr-Gal4/UAS-DUSP31*^*dsRNA*^, *UAS-Rpr/UAS-Dark*^*dsRNA*^ background led to complete suppression of the apoptotic phenotype (Ae and Ae’). Conversely, inactivation of *DUSP31* was not sufficient to suppress the phenotype caused by overexpression of *Rpr* and *Dronc* dsRNA (Ag and Ag’, *Gmr-Gal4/UAS-DUSP31*^*dsRNA*^, *UAS-Rpr/UAS-Dronc*^*dsRNA*^) compared with *Dronc* inactivation alone (Af and Af’, *Gmr-Gal4/+*, *UAS-Rpr/UAS-Dronc*^*dsRNA*^). **(B)** Control with an additional DUB, *Ubpy.* Inactivation of *Ubpy* did not affect the *Rpr*-induced phenotype (Ba, *Gmr-Gal4/UAS-Ubpy*^*dsRNA*^, *UAS-Rpr/+* versus Ab). *Ubpy* inactivation did not suppress the phenotype in fly eyes caused by overexpression of both *Rpr* and dsRNA of *Dark* (Bc, *Gmr-Gal4/UAS-Ubpy*^*dsRNA*^, *UAS-Rpr/UAS-Dark*^*dsRNA*^) compared with the control (Bb, *Gmr-Gal4/+*, *UAS-Rpr/UAS-Dark*^*dsRNA*^).

Next it was assessed whether *DUSP31* deficiency was sufficient to alter apoptosis induced by RHG proteins or IAP antagonists acting upstream of Dark and DIAP1. Overexpressing one copy of *Grim* (*Gmr-Gal4/+*, *Gmr-Grim/+* genetic background) caused depigmentation, mild roughness, and slight reduction in the size of fly eyes (Figure 3Ab versus 3Aa) [29, 40]. As expected, *DUSP31* dsRNA strongly suppressed *Grim*-induced apoptosis in the eye (Figure 3Ac). In controls, inactivation of *Dark* strongly suppressed the *Grim*-induced phenotype (Figure 3Ad). On the other hand, hetero- and homozygous *DUSP31*^*KG04149*^ alleles were found to mildly but substantially suppress the strong apoptotic phenotype induced by overexpression of two copies of *Gmr-Grim* constructs (*Gmr-Gal4/+*, *Gmr-Grim/Gmr-Grim* genetic background) (Figure 3Bb and 3Bc) compared with the wild type background (Figure 3Ba). This observation is consistent with the notion that *DUSP31* function is required to positively regulate apoptotic signals induced by one of RHG proteins, possibly involving stabilization of some proapoptotic factor. Interestingly, dsRNA and heterozygous *DUSP31*^*KG04149*^ alleles of *DUSP31* do not affect the *Reaper*-induced eye phenotype (*Gmr-Gal4/+, UAS-Rpr/+*) (Figure 4Ac versus 4Ab). In addition, similar to the strong *Gmr-Grim* phenotype, *DUSP31*^*dsRNA*^ and heterozygous *DUSP31*^*KG04149*^ alleles could only mildly suppress the apoptotic phenotype induced by *Gmr-Gal4/UAS-DIAP1*^*dsRNA*^, as seen by a slight increase in eye size (Supplementary Figure 1). This data suggests that *DUSP31* plays a role as a fine-tune regulator of proapoptotic factors because strong signals induced by *Rpr*, doubled amounts of *Grim*, or *DIAP1* activity removal (Supplementary Figure 1) caused robust activation of Dark and Dronc. This is also consistent with observations that inactivation of DIAP1 caused caspase-independent cell death, and DIAP1 may be involved in ubiquitination of Dark itself [15]. Dark is constitutively active and triggers activation of Dronc that is not bound by DIAP1. It has also been shown that excessive amounts of Dronc and Dark are degraded through a feedback inhibition mechanism mediated by the catalytic activity of Dronc, as well as by the C-terminal of Dark and DIAP1 [32]. Although the mechanism of Dark degradation is not clear, it likely involves ubiquitination. It has also been shown that overexpression of *DUSP31* in S2 cells does not affect the levels of DIAP1 expression nor its stabilization [38]. Thus this suggests that *DUSP31* acts independently of DIAP1, likely via association with *Grim*-induced signals, and regulates downstream Dark/Dronc proapoptotic transduction sites. Altogether, these results suggest *DUSP31* functions as a regulator of the apoptosome complex downstream of RHG proteins and DIAP1.

### *DUSP31* is involved in positive regulation of *Dark*

The above experiments suggest that *DUSP31* may be involved in regulation of *Dark* function. As seen by residual and mild eye roughness, inactivation of *Dark* by dsRNA did not completely suppress *Rpr*-induced cell death (Figure 4Ad and 4Ad’) compared with wild type and *Rpr*-mutant eyes (Figure 4Aa), suggesting residual Dark activity in *Gmr-Gal4*, *UAS-Rpr*, *UAS-Dark*^*dsRNA*^ mutants. If *DUSP31* protects Dark from degradation, then inactivating *DUSP31* should further suppress Dark-mediated cell death. In agreement with this prediction, inactivation of *DUSP31* on this genetic background completely suppressed cell death in the eye (*Gmr-Gal4*, *UAS-Rpr*, *UAS- Dark*^*dsRNA*^, *UAS-DUSP31*^*dsRNA*^) (Figure 4Ae, 4Ae’ versus 4Ad, 4Ad’). As a control, *Ubpy* DUB dsRNA did not suppress apoptosis in *Gmr-Gal4*, *UAS-Rpr*, *UAS- Dark*^*dsRNA*^, *UAS-Upby*^*dsRNA*^ genetic background (Figure 4Bc versus 4Bb). This suggests that *DUSP31* preferably acts as a positive regulator of Dark. To substantiate this idea further, a similar experiment was performed in which *Dronc* (instead of *Dark*) was inactivated in the *Gmr-Gal4/UAS-Rpr* background (*Gmr-Gal4*, *UAS-Rpr*, *UAS- Dronc*^*dsRNA*^, *UAS-DUSP31*^*dsRNA*^). Similarly, reduction of *Dronc* levels led to strong but not complete suppression of *Rpr*-induced apoptosis characterizing by roughness in the eye (Figure 4Af versus 4Aa). In fact, if this residual activity of *Dronc* can be regulated by *DUSP31*, then it is possible that complete removal by inactive *DUSP31* would completely suppress apoptosis, as shown for *Dark*. However, in contrast to the former, inactivation of *DUSP31* on *Gmr-Gal4*, *UAS-Rpr*, *UAS- Dronc*^*dsRNA*^, *UAS-DUSP31*^*dsRNA*^ background did not suppress the residual apoptosis (Figure 4Ag, 4Ag’ versus 4Af, 4Af’). Thus, this genetic data strongly suggest that *DUSP31* acts as a modifier regulating Dark activity.

**Figure 5:**
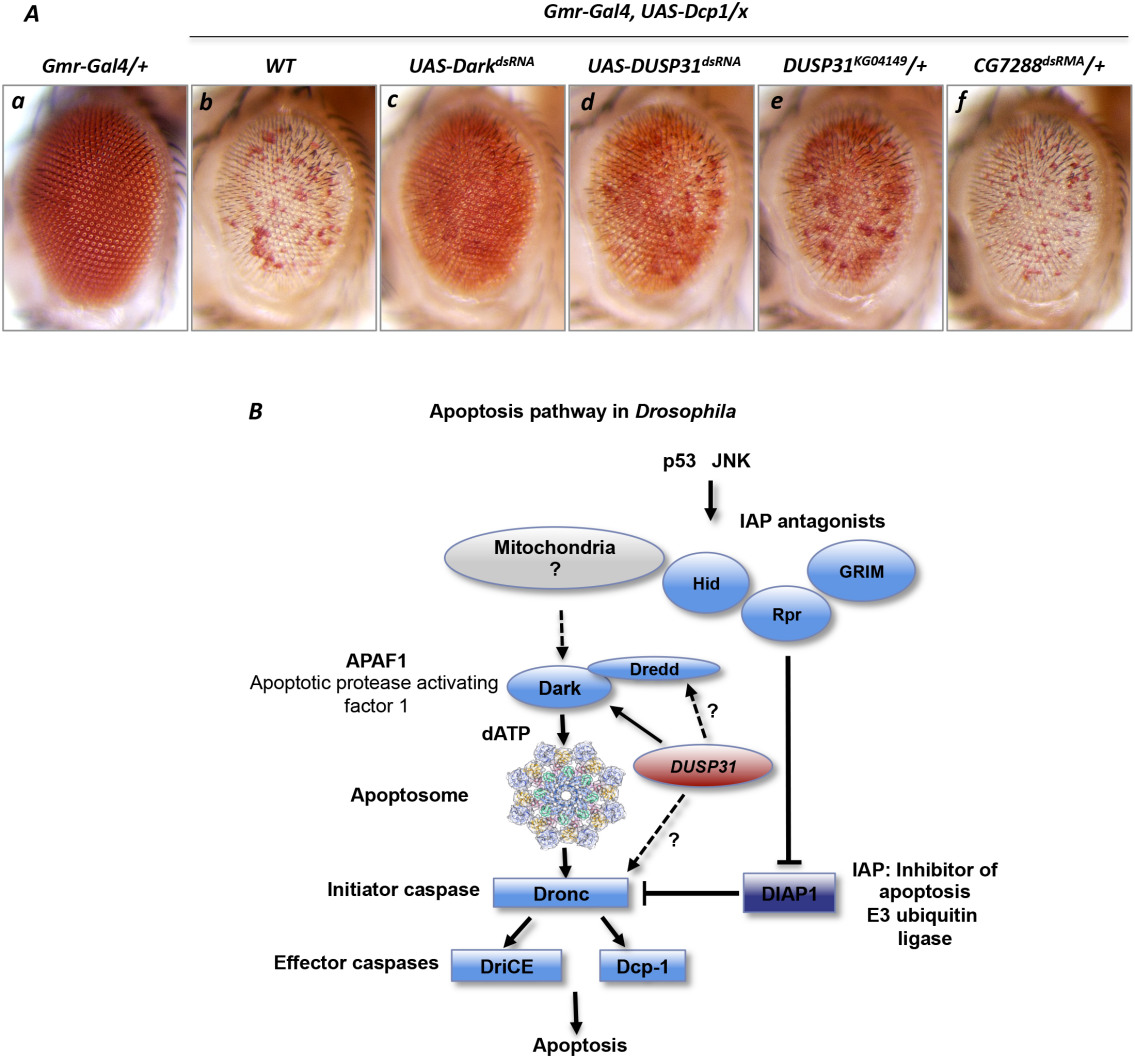
Inactivation of *DUSP31* suppressed the apoptotic phenotype caused by *Dcp-1* overexpression in *Drosophila* eyes Overexpression of *Dcp1* induced apoptosis in fly eyes manifested as depigmentation of a vast majority of eye facets (**Ab**, *Gmr-Gal4/+*, *UAS-Dcp1/+*) compared with *Gmr-Gal4/+* control eyes **(Aa)**. dsRNA-mediated inactivation of *Dark* (**Ac**, *Gmr-Gal4/UAS-Dark*^*dsRNA*^, *UAS-Dcp1/+*), *DUSP31* (**Ad**, *Gmr-Gal4/UAS-DUSP31*^*dsRNA*^, *UAS-Dcp1/+*), and the heterozygous *DUSP31* allele (**Ae**, *Gmr-Gal4/UAS-DUSP31*^*KG04149*^, *UAS-Dcp1/+*), but not dsRNA-mediated inactivation of *CG7288* (**Af**, *Gmr-Gal4/UAS-CG14619*^*dsRNA*^, *UAS-Dronc/+*), suppressed the apoptotic *Dcp-1* phenotype in eye photoreceptor cells (Ab, *Gmr-Gal4/+*, *UAS-Dcp1/+*). **(B)** Schematic representation of the *Drosophila* apoptosis pathway. The proapoptotic function of *DUSP31* is associated with positive regulation of the Dark apoptosome protein or probably with apical caspases Dronc and Dredd (indicated by arrows).

On the other hand, to exclude the possibility that *DUSP31* stabilizes downstream effector Death caspase-1 (Dcp-1), the presence of *DUSP31* deficiency upon *Dcp-1* overexpression in the eye was analyzed. Overexpression of *Dcp1* with *Gmr-Gal4* caused a robust apoptotic phenotype characterized by vast depigmentation in the eye (Figure 5Ab) [44–46] similar to the phenotype induced by overexpression of mutant *Dronc*^*S130A*^. Surprisingly, inactivation of *Dark* also suppressed the *Dcp-1* overexpression phenotype (Figure 5Ac versus 5Ab). Accordingly, inactivation of *Dark* was not predicted to affect the phenotype caused by overexpression of the downstream *Dcp-1* enzyme. However, such was not the case; this can be explained by the dependency of *Dcp-1* activation upon the activity of *Dark* and the apoptosome, which are probably activated via a positive feedback loop upon *Dcp-1* overexpression. This notion is consistent with a previous report showing that an effector caspase can activate the Grim protein located upstream in the pathway [42]. Importantly, both *DUSP31* alleles phenocopied the *Dark* phenotype in *Gmr-Gal4/+*, *UAS-Dcp1/+* genetic background, thereby suppressing the *Dcp-1* overexpression phenotype (Figure 5Ad and 5Ae). In controls, dsRNA-mediated inactivation of another DUB, *CG7288*, did not suppress the *Dcp-1* overexpression phenotype (Figure 5Af). Thus, *DUSP31* likely positively regulates Dark activity in this particular context. Taken together, these genetic data additionally suggest that *DUSP31* acts as a modifier, activating Dark and/or its positive regulators.

To date, the exact mechanism of Dark protein activation and apoptosome complex assembly in *Drosophila* programmed cell death pathways remains poorly understood. In contrast to mammalian Apaf1, Dark associates with cytochrome-c; however, this interaction does not induce apoptosome formation [9]. On the other hand, dATP is important in the formation of this complex in both invertebrate and vertebrate systems [14]. It has also been shown that apical caspase Dredd interacts with Dark, mediating its activation [13]. It has not yet been shown whether Dark can be regulated by any post-translational modifications, including ubiquitination with subsequent proteasomal degradation. Further biochemical studies are required to answer this question. Dredd can be regulated by ubiquitination [47], and it is hypothesized that *DUSP31* antagonizes this process, acting as proapoptotic factor. In the Dark apoptosome complex, the caspase recruitment domains interact with nucleotide-binding domains, leading to conformational changes within the complex to facilitate Dronc recruitment and activation [48–50]. Dronc is constantly ubiquitinated via DIAP1 action. Therefore, the possibility of DUSP31 playing a role during this step and activating the caspase cannot be excluded. Current genetic data described above indicate that *DUSP31* could be involved in the regulation of all of these proapoptotic proteins (summarized in Figure 5B).

*Drosophila DUSP31* encodes ubiquitin C-terminal hydrolase of family 2, called peptidase C19. These DUBs are large proteins ranging in size from 100 to 200 kDa and have two USP catalytic domains, Cys- and His-boxes, and one ubiquitin-like domain [51]. The three predicted and isolated isoforms of DUSP31 have minor differences in a short sequence lying near the Cys-box catalytic center of the protein’s N-terminus (Figure 2B); thus, these isoforms might have functional differences. However, only initial descriptions of the human homologs of DUSP31, USP31 and USP43, are available [33, 52, 53]. For example, it was reported that human USP31 ubiquitinase activity is involved in the regulation of nuclear factor-kappa B activation via components of the tumor necrosis factor signaling pathway [54]. It has also been reported that DUBs can be regulated by allosteric regulation within large multimolecular complexes by phosphorylation, ubiquitination, sumoylation, proteolytic cleavage, and direct stress stimuli, such as reactive oxygen species [33, 55–57]. Recently, several *Drosophila* DUBs were shown to be involved in regulation of apoptosis by stabilizing both anti- and proapoptotic cell death machinery proteins [38, 39, 58, 59]. DUBAI deubiquitinase positively controls the stability of central anti-apoptotic E3 ligase DIAP1. Another *Drosophila* DUB which is homologous to human USP36, called scny/et, functions as a direct or indirect positive regulator of DIAP1 [38, 58]. The DUBA enzyme (also known as OTUD5), belonging to the OTU class of DUBs, possesses Dronc-controlling activity, whose catalytic activity is positively regulated by phosphorylation [39]. The present genetic study identified a proapoptotic function for the deubiquitinase *DUSP31*, which is involved in the positive regulation of Dark/Dronc apoptosome components. This is only an initial step in characterization of this DUB’s function. Further biochemical analyses will decipher the molecular mechanisms of DUSP31 activity and regulation in *Drosophila* programmed cell death, as well as define a possible function for mammalian USP31 in vertebrate apoptosis.

## MATERIALS AND METHODS

### Drosophila stocks and genetics

*w*^*1118*^ flies were used as wild-type controls. Flies were maintained on standard *Drosophila* cornmeal/sucrose/yeast medium at 20, 25, or 29 °C as necessary. Details about the *Drosophila* stocks used are available in Supplementary Tables 1 and 2. The combined and screening fly stocks were made with the two-balancer stock (Stock # 3703, *Drosophila* Bloomington Stock Center). Genotypes of progeny from each cross are described in each figure legend. The modifier genetic screen was developed based on the *UAS/Gal4* expression system [60] using the late developmental driver *GMR-Gal4* in the fly eye at 25°C or other specified temperatures. Micrographs of the eyes of age-matched female progeny are shown.

### Semiquantitative RT–PCR

*DUSP31* transcripts were quantified using a one-step RT–PCR reaction kit (Invitrogen). For each reaction, the *DUSP31* forward (5’-GCACCTGAG-CTCAACGGTTAA-3’) and reverse (5’-CTGTTGTTGCACCGGATGTGA-3’) primers were used in conjunction with 80 ng of total RNA. *Rp49* was amplified with primers described in [61]. RNA was isolated from fly heads with TRIzolR (Invitrogen) and a DNA-free RNA kit (ZYMO Research). The PCR reaction was carried out for 30 cycles, with each cycle consisting of denaturation (94 °C for 30 s), annealing (55 °C for 30 s), and extension (72 °C for 1min).

## SUPPLEMENTARY MATERIALS FIGURE AND TABLES


