# Supplementary material for: Proapoptotic function of deubiquitinase DUSP31 in Drosophila
Source: Oncotarget. 2017 Jul 31;8(41):70452–62. doi: 10.18632/oncotarget.19715 (PMC5642568; doi:10.18632/oncotarget.19715)
Supplement: Supplementary file 1 [file oncotarget-08-70452-s001.pdf]

Proapoptotic function of deubiquitinase *DUSP31* in *Drosophila*

SUPPLEMENTARY MATERIALS

REFERENCES

1. Yang CS, Thomenius MJ, Gan EC, Tang W, Freel CD, Merritt TJ, Nutt LK, Kornbluth S. Metabolic regulation of Drosophila apoptosis through inhibitory phosphorylation of Dronc. EMBO J. 2010; 29:3196-3207.
2. Legan SK, Rebrin I, Mockett RJ, Radyuk SN, Klichko VI, Sohal RS, Orr WC. Overexpression of glucose-6-phosphate dehydrogenase extends the life span of Drosophila melanogaster. J Biol Chem. 2008; 283:32492-32499.
3. Leulier F, Ribeiro PS, Palmer E, Tenev T, Takahashi K, Robertson D, Zachariou A, Pichaud F, Ueda R, Meier P. Systematic *in vivo* RNAi analysis of putative components of the Drosophila cell death machinery. Cell Death Differ. 2006; 13:1663-1674.
4. Kim YI, Ryu T, Lee J, Heo YS, Ahnn J, Lee SJ, Yoo O. A genetic screen for modifiers of Drosophila caspase Dcp-1 reveals caspase involvement in autophagy and novel caspase-related genes. BMC Cell Biol. 2010; 11:9.

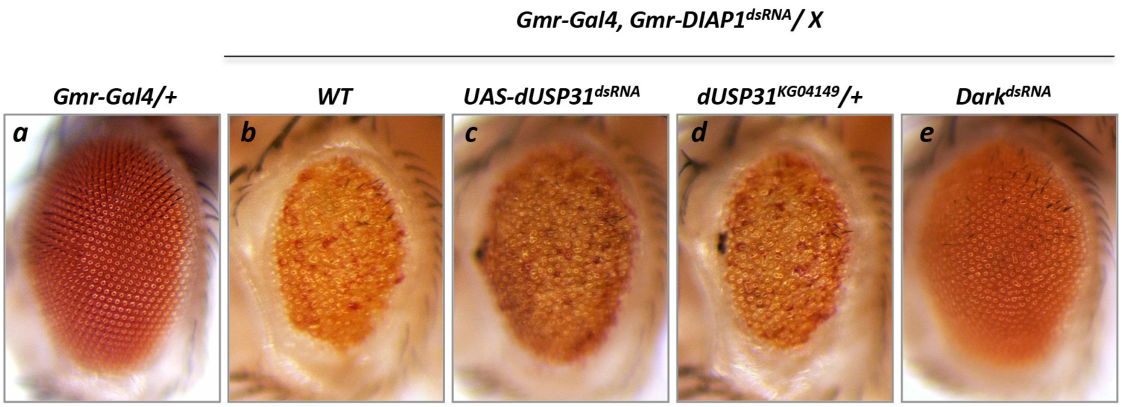

Supplementary Figure 1: Inactivation of *dUSP31* causes slight suppression of *DIAP1* loss-of-function phenotype. Inactivation of *DIAP1* by its dsRNA in fly eye (**b**, *Gmr-Gal4/+, UAS-DIAP1^{dsRNA}/+*) causes apoptosis as seen by significant reduction in size of eye and depigmentation if compare with wild type eye (**a**, *Gmr-Gal4/+*). dsRNA-mediated (**c**, *Gmr-Gal4/UAS-dUSP31^{dsRNA}, UAS-DIAP1^{dsRNA}/+*) inactivation of *DUSP31*, and at lesser extent its heterozygous (**d**, *Gmr-Gal4/UAS-dUSP31 ^{KG04149}, UAS-DIAP1^{dsRNA}/+*) allele can slightly suppress apoptosis phenotype induced by inactivation of *DIAP1* (b, *Gmr-Gal4/+, UAS-DIAP1^{dsRNA}/+*). In a positive control, the inactivation of *Dark* (**e**, *Gmr-Gal4/UAS-Dark^{dsRNA}, UAS-DIAP1^{dsRNA}/+*) can almost completely suppress the *DIAP1* loss of function phenotype.

Supplementary Table 1: dsRNA lines used in the genetic screen

Gene ID	Human homologs	UAS-dsRNA strains	*Dronc*-induced phenotype	*Dronc*^{S130A}-induced phenotype
dUSP31/CG30421	USP31, 43	VDRC/103553; VDRC/33727	+++	++
CG8494	USP20	VDRC/110250; VDRC/28910	-	-
CG7288	USP39	NIG/7288R-1; NIG/7288R-2	-	-
CG7023	USP46, 12	VDRC/100586	-	-
Ubpy/CG5798	USP8	VDRC/107623	--	--
CYLD/CG5603	CYLD	VDRC/101414; NIG/5603R-1	-	-
scny/et/CG5505	USP36	VDRC/105989; VDRC/11152	--	-
CG5384	USP14	VDRC/110227	-	-
CG4165	USP45	NIG/4165R-1; NIG/4165R-2	-	-
CG4968	OTUB1	VDRC/21978	-	-
USP7/CG1490	USP7	VDRC/110324	--	-
DUBAI	USP35	VDRC/28960	-	-
Usp2/CG14619	USP2	NIG/14619R-1, 14619R-1	--	-
CG4751	MPND	VDRC/45530, 26623	-	-
USP1/CG15817	USP1	VDRC/100992	--	-
faf/CG1945	USP9X	NIG/1945R-1, 1945R-1	-	-
DmUSP5/CG12082	USP5	VDRC/17569; NIG/12082R-1	-	-

Column 4 and 5 indicates how particular dsRNA suppresses *Dronc*- or *Dronc[S130A]* induced apoptosis phenotype in the eye, respectively. VDRC, Vienna Drosophila RNAi Center; NIG, Fly stocks of National Institute of Genetics, Japan; +++, strong suppression; +, mild suppression; -- some enhancement; L, lethal; -, no significant change.

Supplementary Table 2: List of *Drosophila* stocks

Fly strains	Source
UAS-Dronc^{WT2}	BDSC/56197
UAS-Dronc^{S130A}	BDSC/56513, 56512
dUSP31/CG30421^{KG04149}	BDSC/13275
UAS-rpr	BDSC/5823, 5824
GMR-Gal4	BDSC/9146
GMR-grim	BDSC/9923
UAS-G6PD	[1, 2]
DIAP1^{4}/th^{4}	BDSC/5053
UAS-DIAP1	BDSC/6657
UAS-DIAP1^{dsRNA}	[3]
UAS-Dcp1	[4]
UAS-Dark^{dsRNA}	BDSC/100405, NIG/35122

BDSC, Bloomington *Drosophila* Stock Center; NIG, Fly stocks of National Institute of Genetics, Japan.
